# Cultivation and Characterization of *Cynara Cardunculus* for Solid Biofuels Production in the Mediterranean Region

**DOI:** 10.3390/ijms9071241

**Published:** 2008-07-15

**Authors:** Panagiotis Grammelis, Anastasia Malliopoulou, Panagiotis Basinas, Nicholas G. Danalatos

**Affiliations:** 1 Institute for Solid Fuels Technology and Applications/Centre for Research & Technology Hellas, 4^th^km N.R. Ptolemaida-Kozani, 50200 Ptolemaida, Greece. E-Mail: grammelis@certh.gr; 2 Department of Agriculture Crop Production and Rural Environment, School of Agricultural Sciences, University of Thessaly, Fytokou Street, N. Ionia, 348446 Volos, Greece. E-Mail: danal@uth.gr

**Keywords:** energy crops, thermogravimetry, devolatilization, combustion, cynara cardunculus

## Abstract

Technical specifications of solid biofuels are continuously improved towards the development and promotion of their market. Efforts in the Greek market are limited, mainly due to the climate particularity of the region, which hinders the growth of suitable biofuels. Taking also into account the increased oil prices and the high inputs required to grow most annual crops in Greece, cardoon (*Cynara cardunculus* L.) is now considered the most important and promising sources for solid biofuel production in Greece in the immediate future. The reason is that cardoon is a perennial crop of Mediterranean origin, well adapted to the xerothermic conditions of southern Europe, which can be utilized particularly for solid biofuel production. This is due to its minimum production cost, as this perennial weed may perform high biomass productivity on most soils with modest or without any inputs of irrigation and agrochemicals. Within this framework, the present research work is focused on the planning and analysis of different land use scenarios involving this specific energy crop and the combustion behaviour characterization for the solid products. Such land use scenarios are based on quantitative estimates of the crop'sproduction potential under specific soil-climatic conditions as well as the inputs required for its realization in comparison to existing conventional crops. Concerning its decomposition behaviour, devolatilisation and char combustion tests were performed in a non-isothermal thermogravimetric analyser (TA Q600). A kinetic analysis was applied and accrued results were compared with data already available for other lignocellulosic materials. The thermogravimetric analysis showed that the decomposition process of cardoon follows the degradation of other lignocellulosic fuels, meeting high burnout rates. This research work concludes that *Cynara cardunculus*, under certain circumstances, can be used as a solid biofuel of acceptable quality.

## 1. Introduction

The production of agricultural biomass and its exploitation for energy purposes can contribute to alleviate several problems, such as the dependence on import of energy products, the production of food surpluses, the pollution provoked by the use of fossil fuels, the abandonment of land by farmers and the connected urbanization. Despite the improved technology in the agricultural sector, the economic feasibility of biomass crops is still uncertain in many European countries under the current market conditions. In general, a substantially greater profit is required for the farmers to change their traditional cultivation with a new one for energy production. This could be successful by introducing crops that require particularly lower inputs. A perennial crop well adapted to the prevailing environmental conditions, well competitive to weeds and with minimal needs to nitrogen and other nutrients would be a very good choice in that respect. This is the reason for the cultivation of cardoon, one among the toughest weeds, for bio-fuel production.

Cardoon (*Cynara cardunculus* L.), also known as Spanish thistle artichoke, is a perennial crop of Mediterranean origin [1], well adapted to the xerothermic conditions of southern Europe [2], typical conditions of arid and semi-arid areas of the Mediterranean environment. It is a multipurpose crop that can be utilized as a raw material in paper pulp industry, as forage in winter time but most importantly as solid and/or liquid biofuel in bio-energy sector [3]. Moreover, the extraction of pharmacological active compounds from Cardoon is also a potential application of the crop, whereas three important products arise; inulin in the roots of cardoon [4, 5] and cynarin and silymarin [6]. The latter two are bitter-tasting compounds, which are found in the leaves, and improve liver and gall bladder function, and stimulate the secretion of digestive juices, especially bile, and lowers blood cholesterol levels.

Cardoon'sgrowth starts after the first rains in autumn and continues during winter and spring until the beginning of summer when soil moisture drops at very low levels and the aerial part of the plant dries out, and the crop is harvested almost dry in the period July-August, avoiding soil compaction risks. Cardoon yields fluctuate from 9 to 26 t ha^−1^ in south Europe, following the variation of the annual precipitation [3, 7–9]. Information on the growth and biomass productivity of cardoon under different soil-environmental conditions is generally limited. Many trials with cardoon were carried out in Greece in the last decade showing that this crop may produce 12 to 16 t d.m. ha^−1^ under rainfed conditions depending on the winter and spring rainfall. Biomass yields in excess of 25 t ha^−1^ are feasible under supplemental irrigation, i.e. 2–3 applications in April-May, when water availability is higher. This is in agreement with other data obtained in the Mediterranean basin [4, 10].

Cardoon'sgrowth and productivity in a series of field experiments varied out in the last decade in Greece are presented in this work. Furthermore, the experimental results from the combustion behaviour of two different samples of cardoon are described. Namely, the selected samples are subjected to standard proximate and ultimate analysis and measurement of their calorific values. Devolatilization and char combustion experiments are realized in a Thermogravimetric Analyzer aiming to export effective conclusions on the behavior of the specific biofuel during the two combustion stages. The accrued results are compared with data on the combustion behaviour acquired for other solid biofuels.

## 2. Experimental Section

### 2.1. Materials and Methods

A number of field experiments have been carried out in Thessaly plain (Experimental Farm of the University of Thessaly, Velestino), Central Greece during the last decade (Table 1). Due to the encouraging results, field experiments have been expanded to west and northern Greece, i.e. Agrinion plain and Kilkis area, respectively, in the last three years. The climate in Greece is typical Mediterranean with cool humid winter and rather dry and warm summer. Small climatic variations exist in Thessaly, the largest Greek lowland and the centre of the country'sagricultural production, which is characterized by a more continental climatic character with colder winters and hot summers, whereas in Agrinion plain winter and summer are mild and precipitation is higher throughout the year. Finally, Kilkis’ climate resembles that of Thessaly, with higher precipitation during spring. The study soils are all deep to very deep, moderately fertile to fertile and are classified as Typic Xerohrept, Vertic Xerochrept, and Aquic Xerofluvent in Thessaly plain; Typic Xerofluvent in Agrinion; and Typic Xerochrept in Kilkis [11]. Split-split plot experimental designs were applied in most experiments with studied factors: plant density, nitrogen dressing, levels of weed control, irrigation water depth, etc. In most experiments, the growth and aerial fresh and dry biomass (total and per plant component) were recorded in consecutive distracting samplings throughout the growing period. Seed yield was determined in the final harvest. Based on all data collected and the local socio-economic conditions, economic and energy budgets for cardoon cultivation have been produced at farm level.

In order to carry out the primary characterization of the fuels, a Thermo Finnigan Flash EA 1112 CHNS elemental analyzer was used. Proximate analysis was performed according to the ASTM standards. Specifically, the ASTM D 3173–87, ASTM D 3174–89 and ASTM D 3175–89a were followed for the determination of moisture, ash content and the volatile matter, respectively. Fixed carbon for each sample was determined by subtracting the volatiles and ash content from the initial mass. The high and low heating values were estimated through empirical formulas from the elemental analyzer CHNS.

As concerns the devolatilization processes, two representative samples of cynara'sbiomass, henceforth named as C1 and C2, were used for this devolatilisation and reactivity study. The difference between them is that the called C2 is the only one that includes seeds resulting in a solid biofuel of improved quality. This state will be verified through the obtained results presenting in this research work. After air-drying, the samples were sieved to the desired particle size. In particular, the particle size fractions selected were in the range of 150–250 μm and 250–500 μm. The pyrolysis and char combustion characteristics for both of samples were studied in a non-isothermal TA Instruments Q600 simultaneous TGA-DSC apparatus. All TGA experiments were conducted in an inert (pyrolysis tests) and an oxidative (combustion tests) environment, at ambient pressure and heating rate 20 °C/min. The constant flow rate of 100ml/min was applied both for high purity helium (He) and air (80/20 in N_2_/O_2_), operating as the heat transfer medium [12]. Prior to the heating program, the system was purged with helium for 10 min at 400 ml/min to ensure that the desired environment was established. Aiming to reduce the effects of heat- and mass- transfer limitations small sample weights, about 20 mg, were placed in an open alumina sample pan. Weight loss (TG curves) and the rate of weight loss (DTG curves) were continuously recorded under dynamic conditions, as functions of time or temperature, in the range of 30–1000 °C. Also, the weight precision of the instrument is 0.1 μg.

### 2.2 Kinetic Modeling

The thermal decomposition of cynara cardunculus is characterized by a high yield of low molecular mass compounds at low heating rate. According to many researchers [13–19], the behaviour of biomass under pyrolysis and combustion conditions can be successfully described by the decomposition of its main fractions, i.e. cellulose, hemicellulose and lignin. Despite the fact that the devolatilization processes are complex because of the simultaneous degradation of the three components, these were assumed to decompose individually. Similarly, the kinetic behaviour of cardoon samples as biomass materials has been modeled by several independent, parallel, first-order reactions [12, 14, 20]. The kinetic model and the equations are reported in detail in previous studies [20, 21]. Briefly, the equations that describe the overall rate of conversion for N reactions and the thermal decomposition of the individual components can be described by:

(1) $$-\frac{dm}{dt}=\sum_{i}c_{i}\cdot \frac{da_{i}}{dt}\;i=1,2,3,...,N$$

and

(2) $$\frac{da_{i}}{dt}=A_{i}\cdot \text{exp}(-E_{i}/RT)\;\cdot {\left(1-a_{i}\right)}^{n}$$

where *A_i_*, *E_i_* and *n* are the pre-exponential factor, activation energy for each component *i* and the order of the reaction, respectively. Coefficient *c_i_* expresses the contribution of the partial processes to the overall mass loss, *m_o_* − *m_char_* :

(3) $$c_{i}=m_{0,i}-m_{char,i}$$

where *m_0,i_* and *m_char,i_* are the initial sample mass and the final char yield – normalized with *m_0_* – of component *i*, respectively.

The reaction between a fuel char and oxygen is considered a very complicate process according to many researchers [22–25]. Some of the factors that influence the reaction and appear to be the most important are the various parts of different properties that chars may be composed of and the variety in surface reactivity of chars during their combustion. In order to include the heterogeneous nature of char into the kinetic model, it is assumed to be consisted of a mixture of various components each of which demonstrates different reactivity. Hence, the sample mass, *m*, normalized by the initial sample mass as a function of time is given by the following formula:

(4) $$m(t)=\sum_{1}^{z}c_{j}[1-a_{j}(t)]+m_{\infty }\;[m(0)=1]$$

where, *n* is the number of components, *c_j_* is the fraction of combustibles in component *j*, and *a_j_(t)* is the reacted fraction of component *j* in time *t*. The term *m*_∞_ is the normalized amount of the solid residues (minerals) at the end of the experiment.

A separate equation was used for each component to describe the dependence of the reaction rate on the temperature and fractional burn-off:

(5) $$\frac{da_{j}}{dt}=A_{j}\exp(-E_{j}/RT)g(P_{O2})f_{j}(a_{j})$$

where, *A_j_* is the pre-exponential factor of component *j*, *E_j_* is the activation energy of component *j, g* expresses the effect of ambient gas composition and *f* describes the change of surface reactivity, as a function of the fractional burn off. It has been proved [23, 24, 26] that function *g* is proportional to the partial pressure of oxygen in air and, hence, its value is stated as *g*(*P_O_*_2_) = *P_O_*_2_ and could be included into the pre-exponential factor, while the *f_j_(a_j_)* function was described by:

(6) $$f_{j}(a_{j})={\left(1-a_{j}\right)}^{n_{j}}$$

where *n_j_* is the reaction order ranging from 0 to 2. The method of nonlinear least squares was applied for the optimization of kinetics parameters by minimizing the objective function:

(7) $$O.F.=\sum {\left[{\left(\frac{dm}{dt}\right)}^{\exp}-{\left(\frac{dm}{dt}\right)}^{\text{calc}}\right]}^{2}$$

where *(dm/dt)^exp^* and *(dm/dt)^calc^* is the experimental and calculated differential thermogravimetric curve, correspondingly.

## 3. Results and Discussion

### 3.1. Cardoon Origin and Cultivation

Cardoon (*Cynara cardunculus*), also known as Spanish thistle artichoke, is a perennial very deep-rooted weed of Mediterranean origin [1], well adapted to the xerothermic conditions of southern Europe and Mediterranean climatic type areas. Cardoon'sgrowth starts after the first rains in autumn and continues during winter and spring until the beginning of summer, when soil moisture drops at very low levels, and the aerial part of the plant dries out. Then the crop can be harvested almost dry (<15% moisture content) in the period July-September, so avoiding drying costs (normally 7- >15 €/t) and soil compaction risks. Fast re-growth starts again after the first rains in the following autumn, and crop canopy is very soon fully closed, and so forth. Our field experiments demonstrated that cardoon, as a very competitive weed itself, wouldn’t allow the mutual growth of other weeds, whereas its growth was not affected by pest and diseases, so that its cultivation can be realized without the use of agro-chemicals. Moreover, its deep and effective rooting system takes perfect advantage of the soil'sinherent fertility so that the crop does not need but modest nitrogen dressings only in very poor soils.

Growing during the rainy period, cardoon takes also good advantage of the winter and spring rains, and performs dry biomass yields of 12–16 t ha^−1^ without any irrigation. However, if the crop receives 2–3 irrigation applications from mid-April to late May (when irrigation water is normally still available in many regions), dry biomass yields in excess of 25 t ha^−1^ may be easily attainable. This is in agreement with other data obtained in the Mediterranean basin [4, 10].

### 3.2. Environmental Benefits from Cardoon Cultivation

Besides the obvious environmental benefits of using energy produced from cardoon, cultivation of this crop has direct positive effects on the environment.

Nitrate pollution: Cardoon needs less nitrogen than many other crops. In many field experiments, high biomass yields were attainable under fertilization dressings from 0 up to 50 kg N/ha in shallow and poor soils. Thus, the modest fertilization dressings of cardoon help controlling the nitrate pollution of surface and ground waters in extensive areas where annual crops (cotton, maize, wheat, etc.) are intensively cultivated.

Reduction of agro-chemicals: Due to its great adaptation, cardoon fast (re)growth controls the mutual growth of other weeds in many environments. On the other hand, in all field experiments, no evidence of cardoon suffering by any pest or disease was present. Therefore, cardoon can be cultivated without the use of any agrochemicals, so further reducing the production cost and the environmental risk from the use of these substances.

Water management: As mentioned, cardoon can take perfect advantage of the winter and spring rains and produces quite high biomass yields without any irrigation.

Soil erosion and land desertification: Cardoon starts growing at particularly high rates just after the first rains in October. Soon its canopy is closed and protects the soil from erosion, which is the most important environmental hazard on the sloping lands around the Mediterranean semi-arid zone.

Improvement of soil characteristics: After cardoon'sestablishment, the only field work is harvesting. Thus, cardoon fields do not suffer from soil compaction. The first leaves formed (“rozeta leaves”) fall off creating a humus rich top soil with improved soil physical (soil structure, permeability and infiltration capacity, increased water holding capacity, etc.) and chemical characteristics (increasing organic matter content, cation exchange capacity, available nitrogen, phosphorus, etc.).

### 3.3. Energy Production and Cost

With a gross heating value of the dry biomass measured at 16.5 GJ/t (seed excluded), Table 1, to 18.5 GJ/t (seed included), such yields correspond to 5.7–7.5 oil equivalent (TOE) ha^−1^ for the rainfed and >11.0 toe ha^−1^ for the supplementary irrigated crops, respectively.

Considering the modest inputs (practically soil preparation and sowing once in a 10 years plus annual harvest and transportation to the plant that is estimated at 70–200 €/ha) cardoon may produce the cheapest biofuel comparing to all other bio-energy crops known. Actually the energy production cost is determined at <0.5–1 €/GJ on the farm, 3 €/GJ including the farmers profit (dry biomass sold against 60 €/t in July 2008), and about 3.5–4.0 €/GJ including the cost of pellet production (note: current oil price in Greece 820 €/t or 20 €/GJ). Unlike other biofuels such as bio-ethanol from maize and biodiesel from oilseed rape (energy balances 1/1.3 and 1/2.5 [27]), heat energy produced from cardoon reaches 1:27, thus leading to a revolutionary state of the art. Additionally, with on farm output/input ratios of 3.5–4.5 €/€ cardoon appears to be by far the more interesting than many other crops in Greece and elsewhere and may secure a very good income to the farmers.

Besides agro-pellet production, cardoon biomass contains 15–20% seed which is by 25% rich in oil that can be used for sustainable production of cheap bio-diesel. Finally, cardoon'srich in cellulose and hemi-cellulose biomass may produce considerable amounts of bio-ethanol in the future (second generation biofuels). Based on the above, cardoon is considered as the most important and promising crop for biomass and energy production in Greece in the near future. Cardoon cultivation may partially replace traditional cultivations ensuring a good profit to the farmer (double compared to wheat and to cotton cultivation with present prices, e.g. 70 €/t dry biomass in the entrance of the factory) and producing biofuel of high energy content. Our research shows that is feasible to introduce 200 kha of cardoon cultivation in Greece in the immediate future (one fifth of the cultivated area with winter cereals, or 5% of the total agricultural land) for the production of over 1 million toe. This would result in an increase of the farm income by 150–180 million €, whereas the operation of 40–50 processing plants and the creation of the new markets would create thousands of many new jobs. The solid biofuel (cardoon pellets, briquettes, etc) may reach the end user at prices by 30–40% lower than the oil price, whereas the dependence on the imported fossil fuels will be remarkably reduced.

### 3. 4. Pyrolysis and Kinetic Modeling of the Cardoon Biofuels

Table 2 presents a complete dataset referring to the proximate and ultimate analyses and the evaluation of calorific value (HHV) on dry basis of cardoon samples. In particular, the percentage of volatile matters varies from 70 to 72 wt%, while the fixed carbon content ranges between 13.4–14.6 wt%, respectively. A higher ash content compared to other solid biofuels is observed, which accounts for 7.2 and 6.9 wt%. However, the high ash values are characteristic for cardoon residues, since this varies between 4% and 17% on dry basis [28–30]. Additionally, both of the samples show comparable results with other studies [15, 28–29] in the nitrogen and sulphur content. More specifically, nitrogen is lower than 1.9 wt% on dry basis, while sulphur equals to 0.1 wt% on dry basis. C2 sample presents the highest elemental carbon and hydrogen content resulting to its higher calorific value (HHV) which is approximately 16.5 MJ/kg. The percentage of its nitrogen value is double compared to the C1 sample. These variations are attributed to the different origin of each sample, i.e. stalk or seed. Generally, the composition of the tested cynara samples is comparable to other biomass materials [12, 20–22].

**Figure 2.**
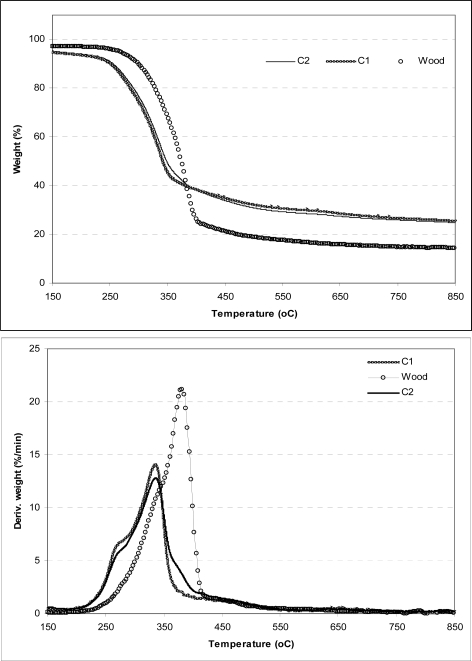
TG (left plot) and DTG (right plot) curves during the pyrolysis of cardoon and wood.

A comparative analysis of the weight loss (TG) and weight loss rate (DTG) curves for cardoon'ssamples at a heating rate of 20 °C/min and particles size fraction of 250–500 μm are presented in Figure 2, respectively. Natural wood and its corresponding thermogravimetric curves have been used to compare the accrued results with artichoke. The shape of the curves indicates that pyrolysis proceeds in almost the same way for all biomasses samples despite the origin for the cynara'ssamples. A more analytical look at the weight loss rate curves reveals a shoulder located at the low temperatures of the degradation of the lignocellulosic samples. It has been reported [13, 16, 19, 22] that the lower temperature shoulder represents the decomposition of hemicellulose present in the biomass material and the higher temperature peak the decomposition of cellulose, whereas the flat tailing section showed above 400 °C corresponds to the degradation of lignin. This curve is more pronounced in the DTG profile of C1 sample indicating its higher hemicellulose content compared to the C2 sample. The shoulder located on the left is less pronounced for C2 sample, while an additional shoulder is present in the region of 380–410 °C. A decrease in the maximum devolatilization rates for cynara'ssamples is obtained when comparing the TG/DTG curves for cardoon residues and wood. Additionally, this rate is attained at earlier temperature for C1 and C2, indicating that cynara is more reactive compared to wood.

The mineral matter is higher in cardoon rather than in natural wood (<1 wt%) and is considered the main component responsible for the low-temperature range of the volatile productions and the subsequent high char yields [15]. However, the degradation of energy crop samples is completed through a wider temperature range. Comparing the given thermogravimetric curves to wood, cellulose and hemicellulose containing in artichoke decompose in lower temperatures, while the third component, lignin, decomposes in the same temperatures as wood.

The main pyrolysis characteristics of the tested C1 and C2 samples, such as initial decomposition temperature (*T_in_*), maximum decomposition rate (*R_max_*) and temperature at that rate (*T_max_*) and total conversion (*C_total_*) are summarized in Table 3.

It is likely that the bigger surface slow down a little the volatiles yielding. On the contrary, the same fraction for both samples presents the higher decomposition rate. In particular, the mentioned rate (Rmax) amounts to values from 11.5–13.8 × 10–2/min in the temperature region of 250–650 °C. The tested samples seem to reach this rate in similar temperatures, specifically, their major weight losses occur between 300–350 °C, which similar to other research studies [31]. The total conversion of the samples for both of the fractions approaches the percentage of 78%. However, the small differences appearing in the devolatilisation characteristics due to the particle size and origin show that they probably differ in reactivity.

**Figure 3.**
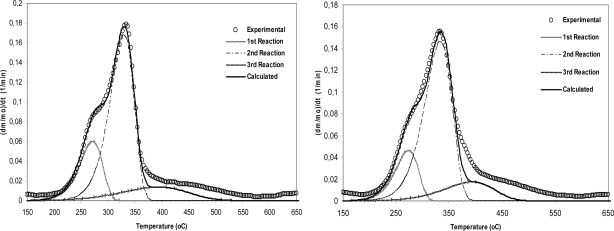
Kinetic evaluation for the pyrolysis of cardoon sample of C1 (left plot) and C2 (right plot) at 20 °C/min by using three independent parallel reactions.

Taking into account the shape of the weight loss rate curves, kinetic modeling of the cardoon pyrolysis was carried out by aggregating the various processes into three reactions, Figure 3.

Finally, the obtained kinetic parameters of cardoon samples are listed in Table 4 accompanying with kinetic data of wood-based material [20] in order to comparative remarks be emerged. On the other hand, a few results on kinetic parameters of cardoon pyrolysis could be found in other research studies [31]. Studying the obtained data, it is more than evident that there are quantitative differences existing between the wood sample and the energy crop. In particular, lower activation energy values are observed for the hemicellulose and cellulose and higher for lignin component during the degradation of cardoon samples. Moreover, the third component, lignin, of C2 sample contributes to the overall mass loss via the highest percentage due to its corresponding increased content demonstrating, consequently, that a considerable part of volatiles is evolved in this region.

### 3.5. Combustion Behaviour of the Fuels

#### 3.5.1. Combustion of the pure fuels

**Figure 4.**
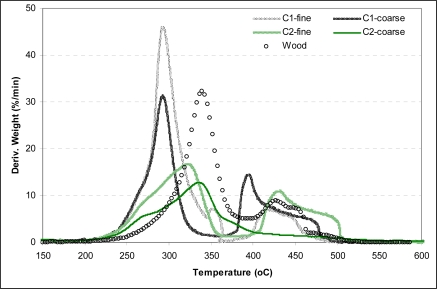
DTG profiles during the combustion of cardoon and wood.

The combustion behaviour of the energy crop'ssamples was also investigated. The knowledge of the possible thermal events during combustion is important for the control and optimization of process. Experimental results of cardoon'ssamples of particles size 250–500 μm combustion are illustrated in Figure 4. These figures depict the reactivity of the material expressed as exactly weight loss and as differential weight loss and temperature. The main combustion characteristics as initial combustion temperature (T*_in_*), maximum combustion rate (R*_max_*), temperature at that rate (T*_max_*), combustion time (*t*) and total conversion (C*_total_*) are reported in Table 4, as well. Studying the TG curves it is shown that the combustion of the fuels tested proceeds in a similar way despite some marked differences. In particular, the combustion of both cardoon samples starts at almost the same temperature (200 °C) even if the combustion of treated sample (C2) starts rather earlier than untreated sample (C1). It is also characteristic that the process starts earlier for the coarse fraction than in the fine fraction (Table 5), despite the fact that particles of smaller diameter accelerate the begging of process due to the higher area available for reaction with oxygen. Nevertheless, it is worthwhile to note that the energy crop'scombustion for both particle sizes is initiated at significantly lower temperatures compared to the paper samples, which start burned at ∼245 °C.

According to the DTG curves, the combustion of cardoon is realized through two temperature regions (a) below 380 °C and (b) from 380 °C to 400 °C. The second stage, which is accompanied by a flat tailing section on the DTG curve, is only observed for sample C1 of both particles size fraction and sample C2 of fine fraction, Figure 6. As demonstrated in previous studies [21] this stage represents the combustion of the char generated during the first stage. The absence of this stage from the DTG curve of the coarse C2 sample is probably attributed to the fact that the combustion of the generated char is taking place in lower temperatures. Therefore, the shoulder in the differential thermogravimetric curve is not clearly visible as a separate stage. Nevertheless, the combustion of the specific sample is continued until 500 °C. Additionally, the C1 sample presents higher peaks located at lower temperatures significant of its higher reactivity during combustion. It is worthwhile to note that the DTG combustion curve of the higher particle fraction C2 sample is located at almost the same temperature region with the main peak of wood. This observation along with the absence of the second curve from the coarse C2 sample shows its slightly better combustion behavior. Considering sample C1, the lower fraction seems to be more reactive in the first temperature region and less in the second one. Thus, the particle size is likely to have an effect on the fuel reactivity.

A comparison referring to the obtained combustion results between cardoon'ssamples demonstrates a higher *R_max_* for the untreated thistle (C1) which mounts to 31.2–46 × 10^−2^/min than C2 sample whose rate accounts for only 16.8–18.6 × 10^−2^/min. Additionally, the maximum combustion rate is reached at earlier temperatures for C1 indicating its higher reactivity, as well. However, more time is needed for sample C1 to finish the combustion process. Finally, as clearly shown in Table 4, the total conversion for both of samples is over 92%.

#### 3.5.2. Char combustion of the fuels

Apart from the devolatilisation processes, the combustion of chars is another thermal treatment method that is essential for the fully understanding of the bio-fuels behaviour during their energy utilization. Despite the widely used one-step reaction [22, 24], the differential thermogravimetric (DTG) data show that combustion of cynara chars is a multistep process. Indeed, weight loss starts at relatively high temperature and occurs to a certain extent also in oxidized environment [25], Table 6.

The char combustion for biomass samples starts at temperature over 290 °C. The only exceptional behaviour is observed for the untreated thistle (C1) char resulted from the pyrolysis of the low particle fraction where the initial combustion temperature is 277 °C. However, this difference is minimal and within the acceptable deviation limits. Additionally, both of the cardoon chars present temperatures where maximum combustion rate occurred (*T_peak_*) lying in the range of 350–375 °C. According to literature [19, 24], these data are comparable with the results obtained by other laboratories using olive kernel (*T_peak_*: 378 °C, *R_max_*: 11.74 × 10^−2^/min) and cotton residue (*T_peak_*: 384 °C, *R_max_*: 8.59 × 10^−2^/min) chars, verifying a similar evolving behaviour. However, the maximum combustion rate of C1 and C2 samples is, at least twice as is for other Biofuels, Table 5, [24].

Apparently, there is a connection between the maximum reaction rate and the temperature where this occurs. This remark has already been referred by many researchers, [32–33] who take *T_peak_* as a measure of combustibility or reactivity [24]. In this sense, the energy crops chars seem to be the more reactive than olive kernel and cotton residue chars, as it showed the highest reaction rate occurred at the lowest temperature, *T_peak_*. Comparing the cardoon samples, the untreated thistle char seems to be the most reactive, since it yields a maximum combustion rate (20.5 × 10^−2^/min) at the earlier temperature, close to 353 °C. In contrast, C2 sample shows the higher maximum combustion rate which accounts for 23.3 × 10^−2^/min and occurred at 368 °C. Also, it is obvious that particle size has also an effect on samples reactivity. Indeed, there is a shift to the left for the fraction of the lower size for both cardoon samples, indicating its higher reactivity. These assertions are verified by the following formula which calculates the reactivity,

(8) $$R=W_{o}^{-1}\cdot (\frac{dw}{dt})$$

where R is the maximum reactivity expressed in mg/h, Wo the initial weight of the char on dry-ash-free basis and dw/dt the maximum rate of fixed carbon loss [34]. Finally, the total conversion of all samples range from 69.4–75.8%.

**Figure 5.**
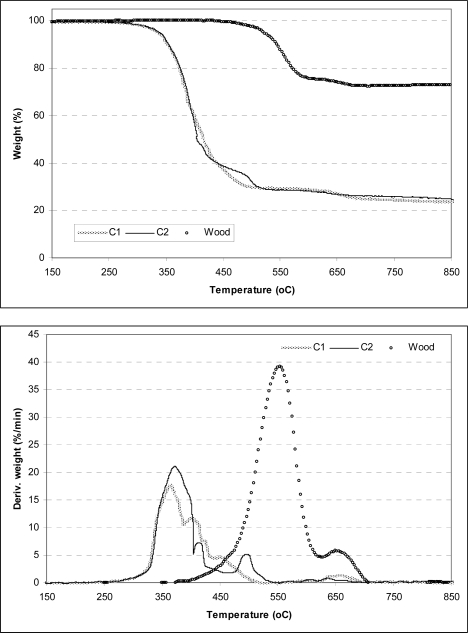
TG and DTG curves during the char combustion of C1, C2 compared to wood.

The weight loss (TG) and the weight loss rate (DTG) curves for the char combustion of energy crop of the bigger particles size fraction compared to wood are illustrated in Figure 5.

As clearly seen in the plots, char combustion for both of cardoon samples is evolved through double and simultaneously overlapped peaks, indicating the relative heterogeneity of char samples. Similar features have been reported in other TG-based studies of char reactivity [22, 24, 35], suggesting that a mixture of two or more reactive components is present.

## 4. Conclusions

Following the remarkable increase of oil price in the last two years, cardoon cultivation and biomass has attracted the interest of large companies towards the construction of medium-scale plants for electrical energy production. Given the heating value of the dry biomass of cardoon (15.5 GJ/t LHV) and that 65 € per t dry biomass is an attractive stimulus of the farmer to cultivate cardoon bring the energy cost at about 60 €/MWh. In Greece, all produced electricity is bought by the National Electric Company against 75 €/MWh (still rather lower than in other EU-25 countries), and this means a gross margin of about 2 million € for a 20 MW_e_ plant only by selling the electricity. The benefit will be by far greater if one considers the heat production and distribution, the selling of pellets for medium-scale industrial uses, the management of the ash for fertilizer use and the rights of CO_2_ emission. But first of all let us think of and protect our environment!

Pyrolysis and combustion behaviour of cynara is investigated in view of the degradation of its main components. Two samples of different particle size were selected and subjected to chemical analyses and thermogravimetric tests. It is found that both samples show a comparable quality to other biomass materials referring to the elemental composition. On the contrary, they present lower calorific values and their higher ash content can accelerate possible formations of slagging and fouling phenomena on various parts of the combustion equipments. However, research efforts that have been carried out aiming at a solid biofuel of improved quality resulted in a combustible material of higher calorific value, i.e. 18 MJ/kg on dry basis. A small further improvement with the reduction of mineral matter of fuel is expected. It is also important to point out that close to zero sulphur values are observed for both of the cardoon samples. This energy crop presents significantly low moisture content, indicating that reduced requirements are needed with regard to their energy exploitation.

Due to their biomass origin, the decomposition process follows the degradation of the lignocellulosic fuels. Pyrolysis proceeds in almost the same way for all samples despite the size or origin - including or not the seed - of the samples. However, an alteration in the particle size caused a small displacement to lower and higher temperatures, respectively, during the combustion process. The data herein provide results of individual cardoon fuels’ characteristics during their thermal conversion which can be used as fundamental information for the prediction of their combustion behaviour. However, additional research is required as concerns the improvement of cardoon properties, especially concerning the energy density, as well as the ash behaviour.

## Nomenclature

*A*: pre-exponential factor [s^−1^]
*C_total_*: Total conversion [% w/w]
*ci*: fraction of volatiles produced by the *i_th_* component
*da_i_/dt*: conversion rate
*dm/dt*: mass loss rate
E: activation energy [kJ/mol]
M: number of parameters involved in the model
*m_0_*: initial dry sample mass [10^−3^ kg]
mchar: final char yield [mg]
*(m_char_)^calc^*: calculated final char yield [10^−3^ kg]
*(m_char_)^exp^*: experimental final char yield [10^−3^ kg]
*m_char,i_*: final char yield of the i component [10^−3^ kg]
*m_i_*: actual sample mass of the i component [10^−3^ kg]
*N*: number of individual reactions
*O.F._DTG_*: objective function
*R*: gas constant [kJ mol^−1^K^−1^]
*R_max_*: Maximum combustion rate [10^−2^/min]
*T*: temperature [°C or K]
*T_in_*: initial combustion temperature [°C]
*T_max_*: temperature at max combustion rate [°C]
*T_max rate_*: temperature at max pyrolysis rate [°C]
*Z*: number of the measured data points
α*_i_*: conversion (reacted fraction)

## Figures and Tables

**Figure 1.**
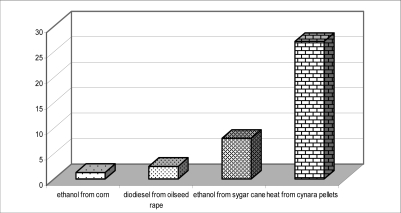
Energy balance for the production of various biofuels [27].

**Table 1.** *Cynara Cardunclulus* L. experimental fields established by the Laboratory of Agronomy of the University of Thessaly during (1999–2007).

Location	Coordinates	Year of Establishment	Studied Factors	Rainfall (mm)	Total dry matter (min–max; in t/ha)
Velestino Magenisia Clay-loam (0.1 ha)	39°12′N 22°14′E 87 m alt	9/3/1999	3 densities (0.6, 1 and 2 pl/m^2^) 2 N-levels (0 and 60 kg N/ha) 4 replicates	≈ 450 mm per year	2^nd^ year: 9.3–15.8 3^rd^ year: 5.7–11.4 4^th^ year: 7.6–11.5
Velestino Magenisia Clay-loam (0.15 ha)	39°12′N 22°14′E 87 m alt	9/9/2004	3 irrigations (350, 100 & 0 mm) 3 N-levels (0, 50 & 100 kg N/ha) 3 replicates	≈ 450 mm per year	1^st^ year: 5.2–6.3 2^nd^ year: 11.1–15.1 3^rd^ year: 12.1–18.6 4^th^ year: ongoing
Palamas Karditsa Loamy 0.1 ha)	39°25′N 22°05′E 105 m alt	13/4/2006	2 irrigations (180 & 0 mm) 3 N-levels (0, 80 & 160 kg N/ha) 4 replicates	≈ 450 mm per year	1^st^ year: 3.7–4.5 2^nd^ year: 27.1–30.8 3^rd^ year: ongoing
Palamas Karditsa Loamy (4 ha)	39°25′N 22°05′E 109 m alt	11/4/2007	2 irrigation (150 & 0 mm) Demonstration field	≈ 450 mm per year	1^st^ year: 2.3–3.2 2^nd^ year: ongoing
Mouries Kilkis Sandy (4 ha)	41°14′N 22°45′E 250 m alt	14/4/2007	2 irrigation (150 & 0 mm) Demonstration field	≈ 600 mm per year	1^st^ year: 1.1–2.9 2^nd^ year: ongoing
Agrinion Sandy clay (1.3 ha)	38°40′N 21°14′E 42 m alt	12/9/2007	Demonstration field	≈ 650 mm per year	1^st^ year: ongoing
Perivolaki Sandy (2.2 ha)	39°12′N 22°14′E 87 m alt	14/3/2007	Demonstration field Weed competition experiments	≈ 450 mm per year	1^st^ year: 2.1–2.9 2^nd^ year: ongoing
Fyteies Kilkis Clay (4 ha)	38°40′N 21°14′E 50 m alt	7/5/2007	2 irrigation (150 & 0 mm) Demonstration field	≈ 650 mm per year	1^st^ year: <1.5 2^nd^ year: ongoing

**Table 2.** Proximate analysis, ultimate analysis and heating value of cardoon samples.

Samples	Proximate analysis (wt%, as received)				Ultimate analysis (wt%, dry basis)					HHV (MJ/kg, dry basis)
	Moisture	V.M.^A^	Fixed Carbon	Ash	C	H	N	S	O^B^	
**C1**	8.2	70.0	14.6	7.2	40.6	5.5	0.9	0.1	45.0	13.7
**C2**	7.9	71.8	13.4	6.9	43.7	6.0	1.8	0.05	40.9	16.3
**Encinar et al** [28, 30]	-	77.3^C^	14.3^C^	8.4^C^	46.7	4.8	0.7	0.1	47.7	18.2

A Volatile matter,

B By subtracting,

C Oven dry basis.

**Table 3.** Devolatilization characteristics of the samples at heating rate 20 °C/min.

Samples	Particle size (μm)	Initial decomposition temperature (°C)	Max decomposition rate (min^−1^×10^−2^)	Temperature at max decomposition rate (°C)	Total conversion (w/w%)
**C1**	150–250	224	11.5	335	78
	250–500	226	13.8	334	76
**C2**	150–250	223	11.9	336	78.2
	250–500	228	12.8	334	77.4

**Table 4.** Calculated kinetic parameters for the pyrolysis of biomass samples at heating rate 20 °C/min.

Samples		Hemicellulose			Cellulose			Lignin		
	Particle size (μm)	A (min^−1^)	E (kJ/mol)	c (%)	A (min^−1^)	E (kJ/mol)	c (%)	A (min^−1^)	E (kJ/mol)	c (%)
**C1**	150–250	1.7 × 10^10^	108.6	16.9	3.2 × 10^11^	134.1	53.3	4.6 × 10^3^	59.6	19.7
	250–500	9 × 10^10^	115.1	18.9	4.6 × 10^11^	135.7	54.8	2.3 × 10^3^	54.5	24.8
**C2**	150–250	5.4 × 10^6^	73.2	14.5	9.3 × 10^7^	95.2	51.5	4 × 10^3^	60.5	34.2
	250–500	5.8 × 10^10^	113.6	14.8	7.9 × 10^9^	116.8	54.5	6.5 × 10^4^	72.8	31.3
Wood-based material [20]		2.6 × 10^11^	131.1	25.3	3.3 × 10^3^	203.2	64	3.3 × 10^3^	56.7	9.8

**Table 5.** Combustion characteristics of the samples at heating rate 20 °C/min.

Samples	Particle size (μm)	Initial combustion temperature (°C)	Max combustion rate (min^−1^×10^−2^)	Temperature at max combustion rate (°C)	Time (min)	Total conversion (%w/w)
**C1**	150–250	198	46	292	44	92.8
	250–500	196	31.2	291	45	92.6
**C2**	150–250	200	16.8	322	42	93.5
	250–500	188	18.6	318	43	92.9

**Table 6.** Char combustions characteristics of the samples at heating rate 20 °C/min.

Samples	Particle size (μm)	Initial combustion temperature (°C)	Max combustion rate (min^−1^×10^−2^)	Temperature at max combustion rate (°C)	Time (min)	Total conversion (%w/w)
**C1**	150–250	277	20.5	353	45	70.2
	250–500	291	17.4	365	33	75.8
**C2**	150–250	296	23.3	368	36	69.4
	250–500	297	21.1	372	40	74.8
